# A novel nutritional inflammation index for predicting mortality in acute ischemic stroke patients: insights into advanced lung cancer inflammation index from the Medical Information Mart for Intensive Care-IV database

**DOI:** 10.3389/fnut.2024.1408372

**Published:** 2024-07-05

**Authors:** Yongwei Huang, Xiaoyi Wang, Zongping Li, Xiaoshuang Yin

**Affiliations:** ^1^Department of Neurosurgery, Mianyang Central Hospital, School of Medicine, University of Electronic Science and Technology of China, Mianyang, China; ^2^Department of Immunology, Mianyang Central Hospital, School of Medicine, University of Electronic Science and Technology of China, Mianyang, China

**Keywords:** advanced lung cancer inflammation index (ALI), acute ischemic stroke (AIS), mortality, MIMIC-IV, biomarker

## Abstract

**Objective:**

This investigation aimed to delineate the association between the advanced lung cancer inflammation index (ALI) and all-cause mortality (ACM) in individuals experiencing acute ischemic stroke (AIS).

**Methods:**

Drawing on information from the Medical Information Mart for Intensive Care (MIMIC)-IV database, release 2.2, covering the years 2012 to 2019, this research assessed the advanced lung cancer inflammation index (ALI) by factoring in body mass index (BMI), serum albumin levels (ALB), and the neutrophil-to-lymphocyte ratio (NLR). Patients with AIS were identified using codes from the International Classification of Diseases (ICD). To address potential confounding factors, a 1:1 propensity score matching (PSM) method was utilized. The investigation identified the pivotal ALI level impacting patient survival using maximally selected rank statistics. It then examined the effects on short- and long-term ACM through multivariate Cox proportional hazards regression models and Kaplan–Meier (K–M) survival analysis. Additionally, restricted cubic spline (RCS) methods were applied to delve into the linear or nonlinear nature of the relationship between ALI and ACM, with further insights gained from interaction and subgroup analyses.

**Results:**

The cohort comprised 838 AIS patients. Post-PSM, analysis involved 199 matched patient pairs. Adjusted Cox proportional hazard models indicated a significant association of low ALI (<10.38) with increased in-hospital ACM, both before (HR: 1.98; 95% CI: 1.36–2.88; *p* < 0.001) and after PSM (HR: 2.16; 95% CI: 1.32–3.52; *p* = 0.002). Associations of low ALI with elevated risk were consistent across ICU, 30 days, 90 days, and 1 year ACM pre- and post-PSM. Subsequent RCS analysis post-PSM underscored a negative nonlinear relationship between ALI and ACM over both short and long terms, without significant interaction effects across different subgroups for ACM.

**Conclusion:**

In this retrospective cohort study, by utilizing a nationally representative sample of United States patients with AIS, our analysis elucidates a negative correlation between the ALI and ACM in individuals with AIS, underscoring the utility of ALI as a novel, efficacious, and accessible inflammatory biomarker for prognosticating ACM. These results carry profound implications for public health policy and practice. A deeper comprehension of these associations can empower public health practitioners and researchers to devise more targeted interventions and policies, aimed specifically at catering to the distinct needs of the AIS patient population, thereby enhancing their health outcomes. The further research in other races/ethnicity is urgent, particularly before applying these findings in clinical practice.

## Introduction

Stroke, as determined by the Global Burden of Disease Study 2021, constitutes a substantial global public health concern. Regarding global prevalence as a cause of mortality, it occupies the fifth position and is the principal cause of death affecting the nervous system (1, 2). Factors such as the rapid aging of populations and urbanization have accentuated the prevalence of stroke risk factors, thus amplifying the overall stroke burden. China, home to nearly a fifth of the global population, faces the highest incidence of stroke worldwide. The rate of AIS incidence in China has seen a notable increase, rising from 117 cases per 100,000 individuals in 2005 to 145 cases per 100,000 by 2019 (3), highlighting the critical challenge AIS presents in terms of both immediate management and long-term rehabilitation. Consequently, the discovery of effective, non-invasive, and easily accessible biomarkers for predicting the clinical outcomes of AIS patients is crucial. Utilizing such markers could lead to more timely and accurate clinical decisions, enhancing patient recovery and reducing death rates.

In contrast to well-known biomarkers like the systemic immune-inflammation index (4), the geriatric nutrition risk index (5), and the prognostic nutrition index (6), the advanced lung cancer inflammation index (ALI) has emerged as a noteworthy prognostic marker for metastatic non-small-cell lung cancer (mNSCLC). ALI combines factors such as body mass index (BMI), serum albumin (ALB), and the neutrophil-to-lymphocyte ratio (NLR), offering a comprehensive measure of a patient’s systemic inflammatory response and nutritional health (7). This blend of nutritional and inflammatory indicators makes ALI an effective gauge of the systemic impact of inflammation and cachexia caused by cancer, making it a valuable metric for assessing how NSCLC patients might respond to immunotherapy treatments (7–9). Additionally, there is evidence to suggest that ALI values correlate with mortality rates in individuals suffering from non-cancer ailments like type 2 diabetes, hypertension, heart failure, and acute coronary syndrome (10–13).

Considering the critical influence of nutrition and inflammation on the progression of AIS in seriously ill individuals, it is posited that lower ALI scores could signal a higher risk of death from any cause. This study intends to scrutinize the connection between ALI and ACM specifically in the ICU context for patients with AIS, aiming to unveil new perspectives on factors determining clinical outcomes and to pinpoint potential strategies for enhancing patient welfare and survival prospects.

## Materials and methods

### Source of data

This investigation was conducted through a retrospective cohort approach, leveraging the Medical Information Mart for Intensive Care (MIMIC-IV) database (version 2.2) (14). Recognized as a critical asset in critical care research, MIMIC-IV provides an extensive collection of anonymized clinical data related to ICU patient care. It features an enhanced and updated compilation of clinical variables ranging from demographic details to comprehensive physiological data and therapeutic measures. Esteemed as one of the most extensive and widely utilized databases in the realm of intensive care medicine, MIMIC-IV furnishes essential assets for analytical and research endeavors. It is instrumental for the exploration of outcomes in critical care, the development of predictive models, support for clinical decision-making, and a variety of other scholarly activities. Permission to access and use the MIMIC-IV database for this study was obtained from both the Massachusetts Institute of Technology and the Institutional Review Board at Beth Israel Deaconess Medical Center (BIDMC, Boston, MA, United States).

### Ethical considerations and data privacy

In adherence to ethical guidelines and to preserve patient confidentiality, this study utilized data that was rigorously de-identified, ensuring all patient information remained confidential. Yongwei Huang, the principal investigator, obtained certification to access the MIMIC-IV database by completing the “Protecting Human Research Participants” online course provided by the National Institutes of Health (Record ID: 12150448), demonstrating compliance with necessary ethical standards for research involving human subjects. Prior to data extraction, Yongwei Huang received specialized training to ensure conformity with established research protocols and methodologies. The research team meticulously crafted a series of data extraction procedures, which were preliminarily tested to refine their precision and feasibility. Furthermore, to verify the data’s reliability, the study incorporated multiple validation strategies, including an independent audit of essential data points and the utilization of statistical software for conducting consistency evaluations, thereby identifying and rectifying any discrepancies or errors. Owing to the anonymized nature of the dataset, the Beth Israel Deaconess Medical Center’s ethics committee exempted the study from the informed consent requirement.

### Population of the study and extraction of variables

**Figure 1:**
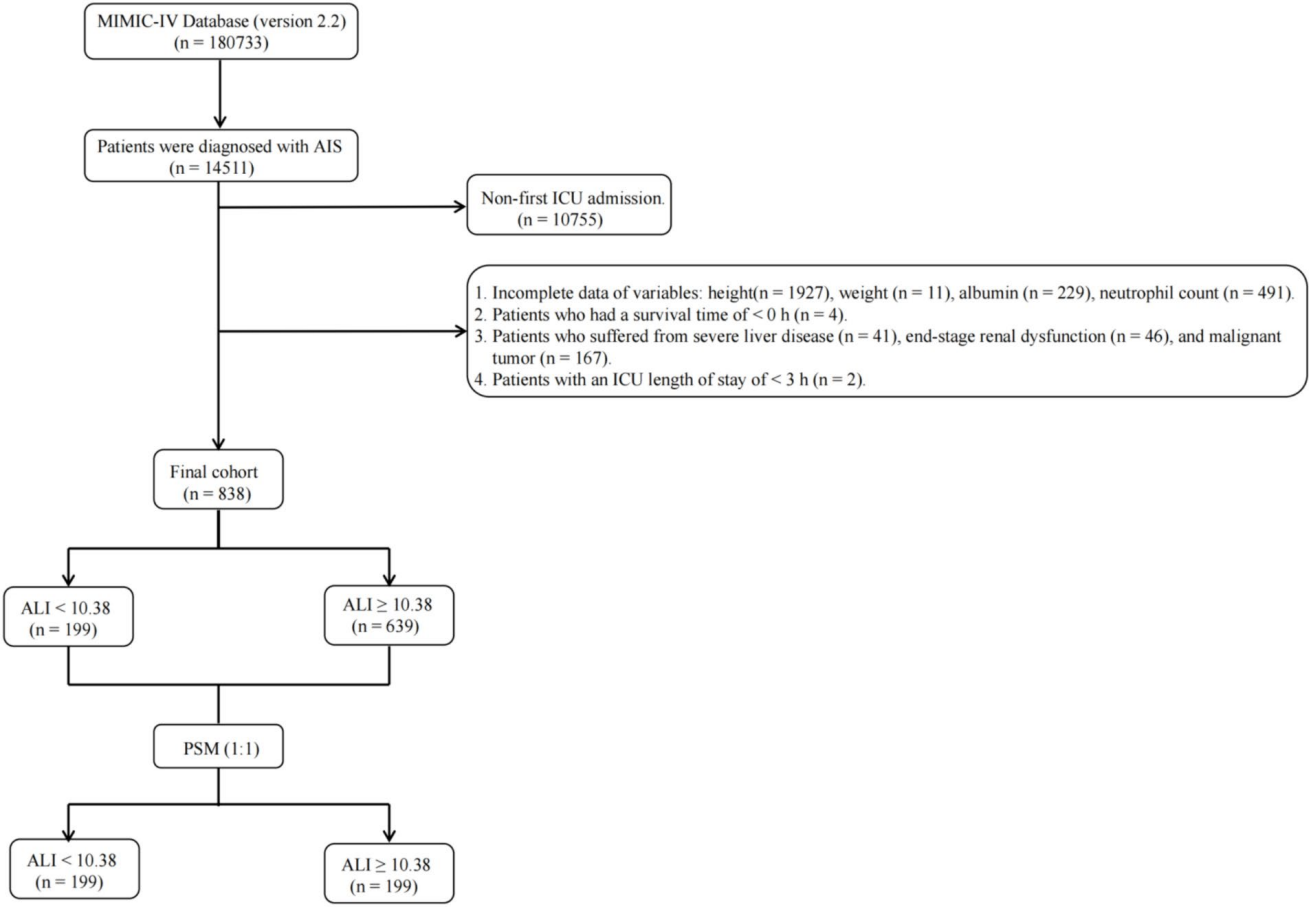
Flow diagram depicting the inclusion and exclusion of participants in the current study. MIMIC, Medical Information Mart for Intensive Care; AIS, acute ischemic stroke; ICU, intensive care unit; ALI, advanced lung cancer inflammation index; PSM, propensity score matching.

The MIMIC-IV database, spanning from 2012 to 2019, includes records for 180,733 individuals. Within this dataset, 14,511 patients were classified as having experienced an AIS, identified through both International Classification of Diseases (ICD)-9 (codes 433, 434, 436, 437.0, 437.1) and ICD-10 (codes I63, I65) criteria. Of these, 10,755 were omitted due to their admission not being the first ICU encounter, narrowing the pool to 3,756 AIS patients. The study focused on adults (≥18 years) and utilized data from their initial ICU admission. Criteria for exclusion included the absence of recorded height (1,927 cases), weight (11 cases), albumin levels (229 cases), and neutrophil counts (491 cases), alongside those with a survival duration of less than 0 h (4 cases), an ICU stay shorter than 3 h (2 cases), or with diagnoses of hematologic neoplasms (0 cases), advanced liver disease (41 cases), terminal renal failure (46 cases), and malignant tumors (167 cases). Following these criteria, a cohort of 838 patients was eligible for final analysis, as depicted in Figure 1.

The focal point of this analysis was the initial blood routine conducted upon ICU admission, designated as the primary exposure variable. Data extraction was performed from the MIMIC-IV database, utilizing SQL queries within a PostgreSQL environment. The extraction encompassed five key areas: demographic information (including age, gender, ethnicity, height, and weight), comorbid conditions (such as hypertension, diabetes mellitus, heart failure, cardiac arrhythmias, peripheral vascular disease, chronic obstructive pulmonary disease, dyslipidemia, previous stroke incidents, and the Charlson comorbidity index), vital statistics (mean and systolic/diastolic blood pressure, heart rate, respiratory rate, SpO_2_), laboratory findings (red and white blood cell counts, hemoglobin, platelets, neutrophils, lymphocytes, ALB, glucose, sodium, creatinine, potassium levels, anion gap, prothrombin, and activated partial thromboplastin times), clinical severity score [Glasgow coma score (GCS), sequential organ failure assessment (SOFA) score, simplified acute physiology score (SAPS)-II, systemic inflammatory response syndrome (SIRS) score, Oxford Acute Severity of Illness Score (OASIS), acute physiology score-III (APS-III)]. Treatments administered (thrombolysis and thrombectomy) and clinical outcomes (length of stay in ICU and hospital, ACM at various intervals) were also documented. To ensure data integrity, variables exhibiting over 20% missingness were excluded. For variables with less than 20% missing data, multiple imputation was applied through a random forest algorithm, facilitated by the “mice” package in R software, to impute missing values, leveraging other available data points.

### Clinical outcomes

The primary outcomes of this investigation encompassed ACM at distinct intervals: during ICU stay, in-hospital, 30 days, 90 days, and 1 year post-ICU admission. Importantly, the determination of mortality was based on incidences of death occurring within these specified periods following ICU admission, providing a temporal context to mortality assessment rather than a static status of life or death at particular time points.

### Propensity score matching

Acknowledging the retrospective nature of this study’s design, which inherently poses risks of selection bias and the introduction of confounders, a propensity score matching (PSM) strategy was employed as a corrective measure. This approach entailed the formulation of a logistic regression model for the generation of propensity scores, subsequently applied to pair patients in a 1:1 ratio. In the PSM process, covariates were selected based on a comprehensive review of previous literature. This pairing was based on a comprehensive array of variables, including but not limited to age, gender, ethnicity, body weight, presence of diabetes mellitus and dyslipidemia, various blood pressure metrics, heart and respiratory rates, SpO_2_, and counts of various blood cell types, alongside serum albumin and glucose levels, and multiple critical care scoring systems. This selection process ensures that the covariates used in PSM are scientifically justified and relevant to the study context. The matching process utilized a nearest neighbor matching algorithm with a caliper width set at 0.1, aiming for minimal deviation in matched pairs. The effectiveness of the PSM in achieving a balanced distribution of baseline characteristics across the groups was assessed through the computation of absolute standardized differences (ASDs). The achievement of ASD values less than 0.10 post-matching was indicative of a successful mitigation of potential biases and confounders, ensuring a balanced comparison between the groups post-PSM analysis.

### Statistical analysis

**Figure 2:**
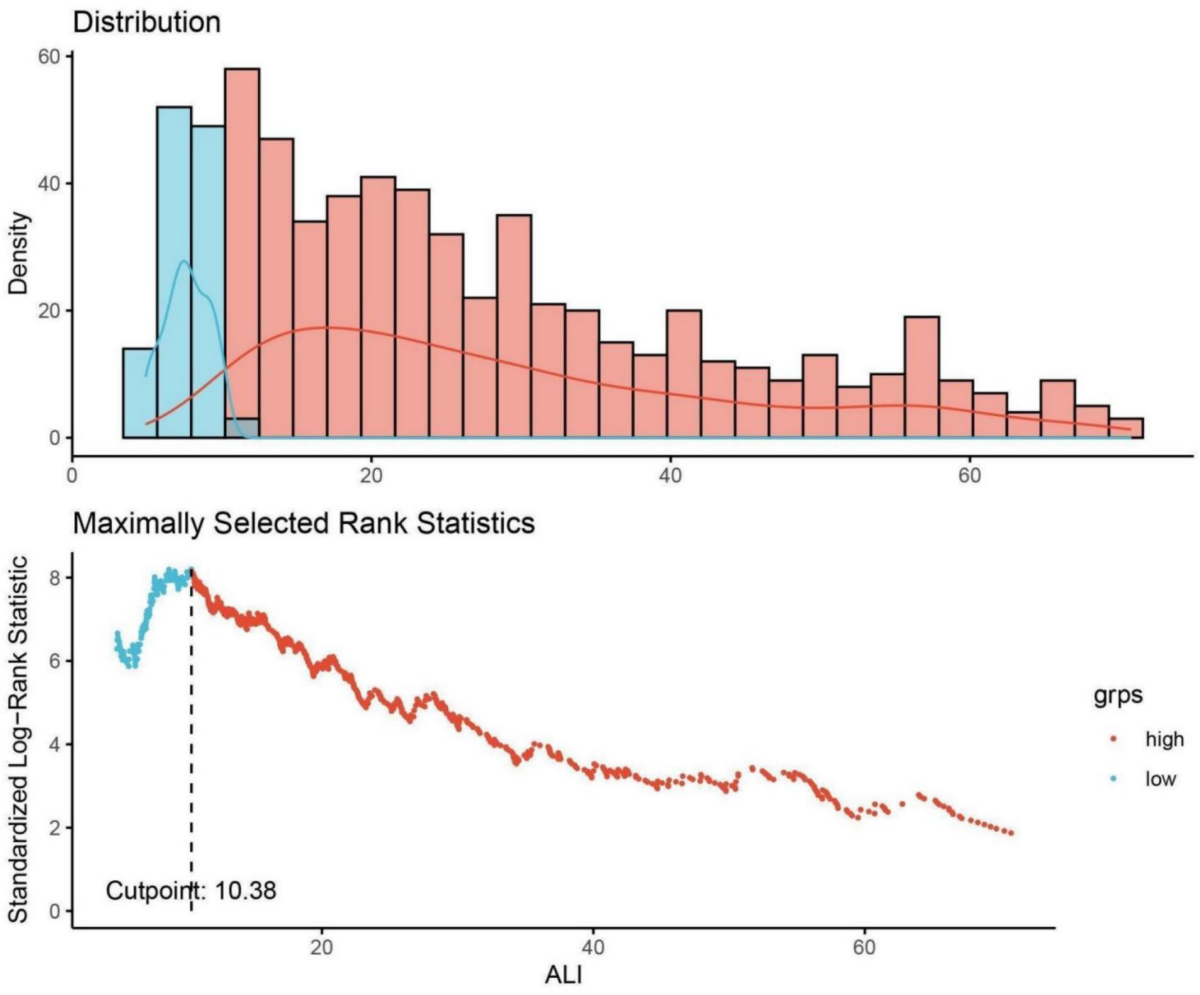
Determination of the ALI cutoff point using maximally selected rank statistics. ALI, advanced lung cancer inflammation index. Standardized log-rank statistic was utilized in the calculation.

In the analytical approach of this study, continuous variables were delineated by their median values accompanied by the interquartile range (IQR), with their disparities evaluated through either the t-test or the Mann–Whitney U-test. Before applying the Mann–Whitney *U* test, we tested the distribution of the continuous variables using the Shapiro–Wilk test for normality. Given that the variables did not follow a normal distribution, the Mann–Whitney *U* test was deemed appropriate. For categorical variables, presentations involved enumerations alongside proportions, and their comparisons were facilitated by either the Chi-square test or Fisher’s exact test. The delineation of the optimal threshold for the ALI for prognostic efficacy in ACM was achieved through the employment of maximally selected rank statistics, setting an optimal cutoff at 10.38. This demarcation led to the bifurcation of ALI into two distinct categories based on this predetermined threshold. The determination of this cutoff, which notably optimized the risk ratio, is illustrated in Figure 2, alongside the distribution of ALI values and the association of ALI <10.38 with ACM outcomes.

To evaluate the stability of hazard ratios (HRs) over time, the study utilized both visual and statistical methodologies to affirm the proportional hazards assumption. Kaplan–Meier curves provided a graphical representation, while the Schoenfeld residuals and Grambsch–Therneau tests offered a formal statistical validation. Given the presence of censored data—participants who did not encounter the event of interest within the study timeline—the Cox regression model incorporated these instances as non-events for the duration of the study period. The temporal metric for analysis was defined from the point of ICU admission to the occurrence or non-occurrence of death at the study’s terminal point.

The study investigated prognostic indicators for mortality following AIS, both in the short and long term, by employing univariate and multivariate analyses within the Cox proportional hazards model. This approach identified significant predictors of ACM, expressed as HRs with 95% confidence intervals (CIs). Additionally, subgroup analyses shed light on how AIS influences mortality differently across various patient groups and comorbidities through a multivariate Cox regression approach with specific stratification. This stratification examined factors such as age (divided into <70 and ≥70 years categories), gender, and the presence of hypertension, diabetes mellitus, and dyslipidemia. The study further divided the AIS variable into quartiles to explore how different levels of the ALI relate to ACM, with a particular focus on comparisons to the highest quartile.

To explore potential non-linear relationships, the analysis incorporated restricted cubic splines (RCSs) to achieve more flexible curve fitting, using generalized additive models for a nuanced examination of ALI’s impact on ACM. This method aimed to identify any threshold effects and the exact point of inflection for ALI’s influence on mortality in AIS patients. We selected the number and placement of knots based on both previous literature and preliminary exploratory analysis. Specifically, knots were placed at the 5th, 35th, 65th, and 95th percentiles of the ALI distribution and confirmed by initial model diagnostics in our data set. This approach ensures robust and reliable fitting of the nonlinear relationship, minimizing the potential for over fitting or under fitting. Statistical testing was two-sided, maintaining a significance level at *p*-values less than 0.05. The data analysis was conducted using R statistical software (version 4.2.2), SPSS Statistics 26, and GraphPad Prism 8, ensuring a comprehensive statistical evaluation.

## Results

### Baseline characteristics of subjects

This investigation encompassed 838 individuals from a cohort of 3,756 patients with AIS who underwent treatment in ICUs. The demographic composition included 484 males (57.76%) and 354 females (42.24%), with a median age of 69 years (IQR: 60–78 years). Participants were stratified into two cohorts according to the ALI threshold identified through maximally selected rank statistics, categorizing them into a group with ALI <10.38 (low ALI) and another with ALI ≥10.38 (high ALI). Prior to implementing propensity score matching, a comparative analysis revealed that the low ALI cohort exhibited lower body weight, decreased prevalence of diabetes mellitus and dyslipidemia, along with elevated averages in blood pressure (both mean and diastolic), heart rate, respiratory rate, and counts of red blood cells, white blood cells, platelets, neutrophils, and lymphocytes. Furthermore, this group demonstrated higher levels of serum ALB, glucose, and sodium, alongside increased scores in several critical care assessment tools including the SOFA, SAPS-II, SIRS, OASIS, APS-III, and the incidence of thrombectomy. Additionally, individuals with ALI <10.38 experienced longer durations of ICU and hospital stays. A detailed comparison of these findings, elucidating the heightened risk of adverse outcomes in patients with lower ALI, is systematically presented in Table 1.

**Table 1:** Baseline characteristics and outcomes of participants before PSM.

Variables	Overall (*n* = 838)	ALI		*p*-value
		<10.38 (*n* = 199)	≥10.38 (*n* = 639)	
*Demographics*				
Age, years	69 (60–78)	69 (56–79)	69 (60–78)	0.56
Men, *n* (%)	484 (57.76)	108 (54.27)	376 (58.84)	0.25
Ethnicity, *n* (%)				<0.001
White	496 (59.19)	99 (49.75)	397 (62.13)	
Black	89 (10.62)	13 (6.53)	76 (11.89)	
Asian	25 (2.98)	2 (1.00)	23 (3.60)	
Others	228 (27.21)	85 (42.71)	143 (22.38)	
Height, cm	1.68 (1.61–1.77)	1.68 (1.60–1.78)	1.68 (1.63–1.77)	0.94
Weight, kg	78.4 (66.5–92.0)	73.5 (60.0–88.8)	80.2 (67.8–93.0)	<0.001
*Comorbidities*				
Hypertension, *n* (%)	444 (52.98)	95 (47.74)	349 (54.62)	0.09
Diabetes mellitus, *n* (%)	298 (35.56)	55 (27.64)	243 (38.03)	0.008
Heart failure, *n* (%)	229 (27.33)	59 (29.65)	170 (26.60)	0.40
Cardiac arrhythmias, *n* (%)	338 (40.33)	74 (37.19)	264 (41.31)	0.30
Peripheral vascular disease, *n* (%)	125 (14.92)	21 (10.55)	104 (16.28)	0.05
Chronic obstructive pulmonary disease, *n* (%)	84 (10.02)	24 (12.06)	60 (9.39)	0.27
Dyslipidemia, *n* (%)	540 (64.44)	89 (44.72)	451 (70.58)	<0.001
Prior stroke, *n* (%)	96 (11.46)	18 (9.04)	78 (12.21)	0.22
Charlson comorbidity index	6 (4–8)	6 (4–8)	6 (5–8)	0.21
*Vital signs*				
Mean blood pressure, mmHg	85 (73–99)	88 (75–100)	83 (73–97)	0.04
Systolic blood pressure, mmHg	127 (108–144)	129 (111–147)	126 (108–144)	0.23
Diastolic blood pressure, mmHg	66 (56–79)	70 (59–84)	65 (54–78)	0.007
Mean heart rate, beats/min	80 (73–93)	88 (77–105)	80 (72–90)	<0.001
Respiratory rate, times/min	18 (15–21)	19 (16–24)	17 (15–20)	<0.001
SpO_2_, %	99 (96–100)	99 (95–100)	99 (97–100)	0.02
*Laboratory parameters*				
Red blood cell, 10^9^/L	3.55 (2.95–4.19)	3.67 (3.11–4.23)	3.47 (2.92–4.14)	0.03
White blood cell, 10^9^/L	11.2 (7.9–15)	12.9 (9.6–17.1)	10.7 (7.6–14.3)	<0.001
Hemoglobin, g/L	11.0 (9.0–12.7)	10.8 (9.0–12.6)	11.0 (9.0–12.7)	0.81
Platelets, 10^9^/L	174 (127–230)	181 (133–261)	173 (126–223)	0.03
Neutrophil count, 10^9^/L	6.87 (4.64–10.35)	11.61 (8.74–14.97)	5.73 (4.30–8.72)	<0.001
Lymphocyte count, 10^9^/L	1.43 (0.90–2.10)	0.69 (0.52–0.99)	1.68 (1.22–2.30)	<0.001
Serum ALB, mg/dl	3.8 (3.4–4.2)	3.4 (2.8–4.0)	3.9 (3.6–4.3)	<0.001
Serum glucose, mg/dl	130 (107–165)	134 (112–174)	129 (105–162)	0.04
Serum sodium, mmol/L	138 (135–141)	139 (135–142)	138 (135–141)	0.08
Serum potassium, mmol/L	4.2 (3.8–4.7)	4.2 (3.8–4.6)	4.2 (3.8–4.8)	0.59
Serum creatinine	0.9 (0.7–1.2)	0.9 (0.7–1.3)	0.9 (0.7–1.2)	0.24
Anion gap, mmol/L	13 (12–16)	14 (12–16)	13 (12–15)	0.16
Prothrombin time, s	13.8 (12.2–16.2)	13.7 (12.1–15.7)	14.0 (12.2–16.3)	0.51
Activated partial thromboplastin time, s	29.6 (26.6–34.7)	28.6 (25.7–34.9)	29.9 (26.8–34.7)	0.07
ALI	21.63 (10.72–41.22)	6.07 (3.51–7.96)	28.86 (18.19–50.67)	<0.001
*Clinical severity scores*				
GCS	15 (15–15)	15 (15–15)	15 (15–15)	0.94
SOFA	1 (0–3)	1 (0–3)	1 (0–3)	0.004
SAPS-II	35 (29–44)	37 (30–48)	35 (28–43)	0.006
SIRS	3 (2–3)	3 (2–4)	2 (2–3)	<0.001
OASIS	33 (27–39)	35 (30–42)	32 (27–38)	<0.001
APS-III	39 (30–53)	44 (34–61)	37 (29–50)	<0.001
*Treatment*				
Thrombolysis, *n* (%)	39 (4.65)	13 (6.53)	26 (4.07)	0.15
Thrombectomy, *n* (%)	29 (3.46)	15 (7.54)	14 (2.19)	<0.001
*Clinical outcomes*				
LOS ICU, day	3.94 (1.96–8.13)	5.29 (2.82–9.95)	3.37 (1.81–7.52)	<0.001
LOS hospital, day	10.13 (5.75–19.33)	12.88 (7.00–22.79)	9.75 (5.58–17.42)	0.002
ICU ACM, *n* (%)	92 (10.98)	40 (20.10)	52 (8.14)	<0.001
In-hospital ACM, *n* (%)	123 (14.68)	55 (27.64)	68 (10.64)	<0.001
30 days ACM, *n* (%)	143 (17.06)	67 (33.67)	76 (11.89)	<0.001
90 days ACM, *n* (%)	189 (22.55)	87 (43.72)	102 (15.96)	<0.001
1 year ACM, *n* (%)	229 (27.33)	100 (50.25)	129 (20.19)	<0.001

ALI, advanced lung cancer inflammation index; SpO_2_, saturation of pulse oxygen; ALB, albumin; GCS, Glasgow coma score; SOFA, sequential organ failure assessment score; SAPS-II, simplified acute physiology score-II; SIRS, systemic inflammatory response syndrome score; OASIS, Oxford Acute Severity of Illness Score; APS-III, acute physiology score-III; LOS, length of stay; ICU, intensive care unit; ACM, all-cause mortality; PSM, propensity score matching.

### Cox regression analyses evaluating the relationship between ALI and ACM in AIS patients before PSM

To investigate the association between the ALI and ACM among AIS patients, this study employed both univariate and multivariate Cox regression analyses with ALI dichotomized. Initial analysis (Model 1), without any adjustments, revealed a significant correlation between a lower ALI (<10.38) and increased ACM risk at various follow-up durations: ICU stay (HR = 1.97, 95% CI: 1.30–2.98, *p* = 0.001), during hospitalization (HR = 2.21, 95% CI: 1.54–3.16, *p* < 0.001), 30 days (HR = 3.16, 95% CI: 2.27–4.38, *p* < 0.001), 90 days (HR = 3.22, 95% CI: 2.41–4.28, *p* < 0.001), and 1 year (HR: 2.73.95% CI: 2.07–3.59, *p* < 0.001), with HRs indicating a heightened mortality risk at each interval. Subsequent adjustment for demographics (age, gender, ethnicity) in Model 2 sustained the association of decreased ALI with elevated ACM risks. A further refined multivariate Model 3, incorporating additional confounders like hypertension, diabetes, heart failure, and interventions (thrombolysis, thrombectomy), alongside blood cell counts and systolic blood pressure, corroborated the independent prognostic significance of lower ALI values for increased mortality risk across the specified time points. These findings are detailed in Table 2.

**Table 2:** Cox regression analyses assessing the association between ALI and ACM in AIS patients before PSM.

Clinical Outcomes	Model 1		Model 2		Model 3	
	HR (95% CI)	*p*-value	HR (95% CI)	*p*-value	HR (95% CI)	*p*-value
**ICU ACM**						
ALI (<10.38)	1.97 (1.30–2.98)	0.001	1.83 (1.20–2.80)	0.005	1.74 (1.12–2.68)	0.01
*ALI (quartile)*						
<10.71	1.73 (0.99–3.02)	0.05	1.59 (0.90–2.80)	0.11	1.46 (0.82–2.62)	0.20
10.72–21.59	0.77 (0.40–1.48)	0.43	0.73 (0.38–1.41)	0.35	0.68 (0.35–1.33)	0.26
21.66–41.22	0.88 (0.44–1.74)	0.70	0.89 (0.44–1.78)	0.74	0.91 (0.45–1.84)	0.80
>41.42	Reference		Reference		Reference	
*p* for trend		0.03		0.08		0.21
**In-hospital ACM**						
ALI (<10.38)	2.21 (1.54–3.16)	<0.001	2.02 (1.40–2.92)	<0.001	1.98 (1.36–2.88)	<0.001
*ALI (quartile)*						
<10.71	2.00 (1.21–3.32)	0.007	1.81 (1.08–3.03)	0.02	1.73 (1.02–2.93)	0.04
10.72–21.59	0.84 (0.46–1.52)	0.56	0.85 (0.46–1.54)	0.59	0.75 (0.41–1.39)	0.36
21.66–41.22	0.98 (0.54–1.79)	0.96	0.96 (0.53–1.75)	0.89	1.03 (0.56–1.89)	0.92
>41.42	Reference		Reference		Reference	
*p* for trend		0.003		0.01		0.04
**30 days ACM**						
ALI (<10.38)	3.16 (2.27–4.38)	<0.001	2.86 (2.04–4.02)	<0.001	2.59 (1.82–3.67)	<0.001
*ALI (quartile)*						
<10.71	3.14 (1.97–5.01)	<0.001	2.80 (1.74–4.50)	<0.001	2.39 (1.47–3.90)	<0.001
10.72–21.59	1.12 (0.65–1.94)	0.68	1.11 (0.64–1.94)	0.71	0.96 (0.54–1.69)	0.88
21.66–41.22	0.99 (0.56–1.75)	0.98	0.97 (0.55–1.72)	0.93	0.96 (0.54–1.69)	0.87
>41.42	Reference		Reference		Reference	
*p* for trend		<0.001		<0.001		<0.001
**90 days ACM**						
ALI (<10.38)	3.22 (2.41–4.28)	<0.001	2.98 (2.22–3.99)	<0.001	2.73 (2.01–3.70)	<0.001
*ALI (quartile)*						
<10.71	3.59 (2.36–5.46)	<0.001	3.23 (2.11–4.95)	<0.001	2.81 (1.82–4.34)	<0.001
10.72–21.59	1.43 (0.89–2.30)	0.14	1.39 (0.86–2.25)	0.18	1.22 (0.75–1.99)	0.42
21.66–41.22	1.03 (0.62–1.71)	0.91	0.98 (0.59–1.64)	0.95	0.92 (0.55–1.55)	0.76
>41.42	Reference		Reference		Reference	
*p* for trend		<0.001		<0.001		<0.001
**1 year ACM**						
ALI (<10.38)	3.06 (2.36–3.98)	<0.001	2.93 (2.24–3.83)	<0.001	2.73 (2.07–3.59)	<0.001
*ALI (quartile)*						
<10.71	3.73 (2.53–5.50)	<0.001	3.46 (2.33–5.13)	<0.001	3.07 (2.05–4.58)	<0.001
10.72–21.59	1.59 (1.04–2.45)	0.03	1.55 (1.00–2.40)	0.05	1.39 (0.90–2.16)	0.14
21.66–41.22	1.14 (0.72–1.81)	0.56	1.09 (0.68–1.73)	0.72	1.03 (0.64–1.64)	0.91
>41.42	Reference		Reference		Reference	
*p* for trend		<0.001		<0.001		<0.001

Model 1: Unadjusted. Model 2: Adjusted age, gender, and ethnicity. Model 3: Adjusted age, gender, ethnicity, hypertension, diabetes, heart failure, thrombolysis, thrombectomy, white blood cell, red blood cell, systolic blood pressure, and SOFA. HR, hazard ratio; CI, confidence interval; ACM, all-cause mortality; ICU, intensive care unit; ALI: advanced lung cancer inflammation index; PSM: propensity score matching; AIS: acute ischemic stroke.

**Figure 3:**
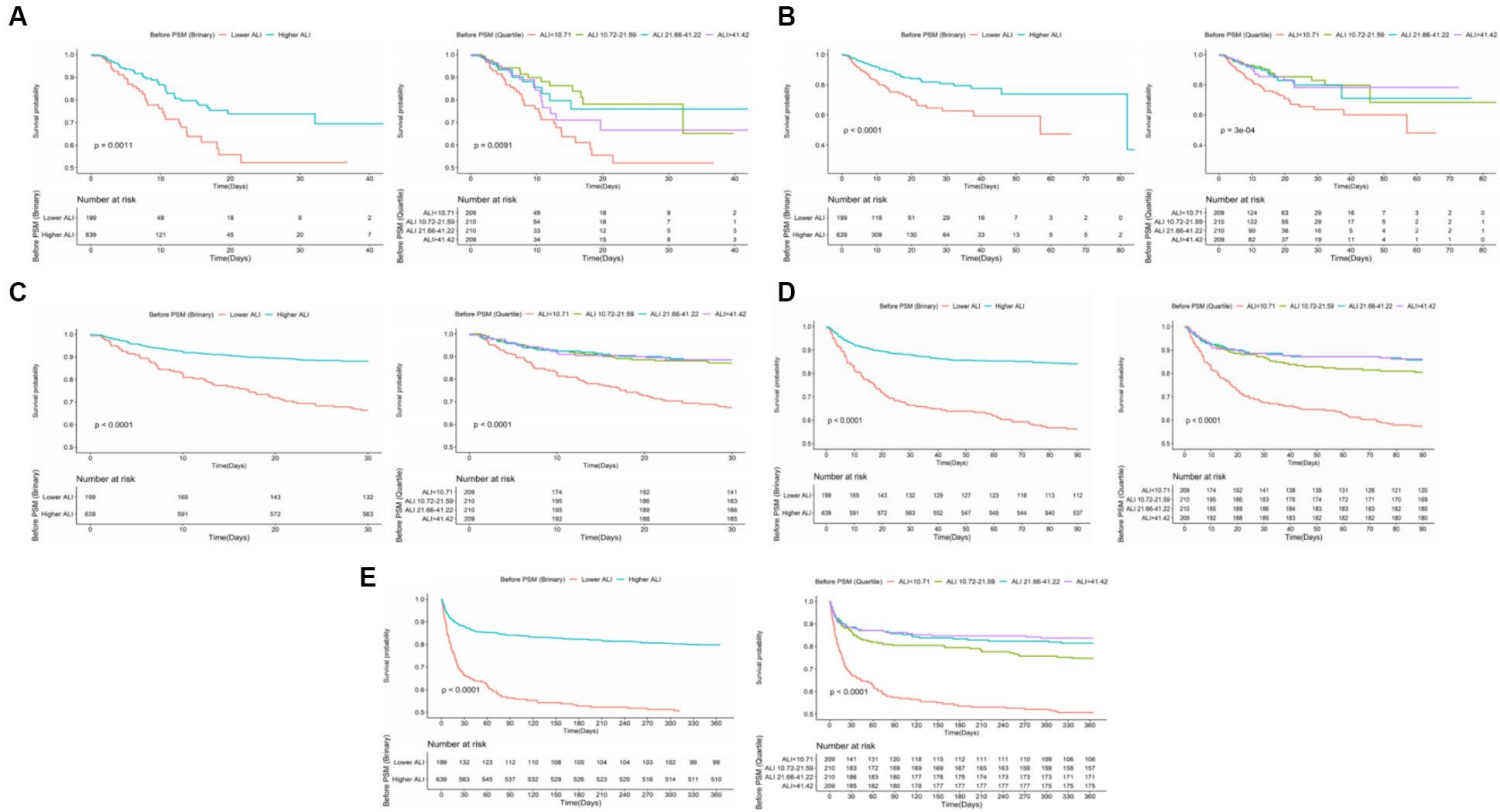
The K–M survival curves of **(A)** ICU, **(B)** in-hospital, **(C)** 30 days, **(D)** 90 days, **(E)** 1 year ACM categorized by binary and quartiles of ALI before PSM. ACM, all-cause mortality; ALI, advanced lung cancer inflammation index; PSM, propensity score matching.

Kaplan–Meier survival curves further validated the disparity in ACM rates between patients with lower and higher ALI scores. Specifically, the survival analysis underscored significantly higher mortality rates at ICU, in-hospital, 30 days, 90 days, and 1 year marks for the lower ALI cohort compared to their higher ALI counterparts, with percentages indicating substantial differences in survival outcomes (20.10% vs. 8.14%, *p* < 0.001; 27.64% vs. 10.64%, *p* < 0.001; 33.67% vs. 11.89%, *p* < 0.001; 43.72% vs. 15.96%, *p* < 0.001; 50.25% vs. 20.19%, *p* < 0.001, respectively). Further insights into these discrepancies are graphically depicted in Figure 3.

### Relationship between the ALI and ACM in AIS patients after PSM

**Figure 4:**
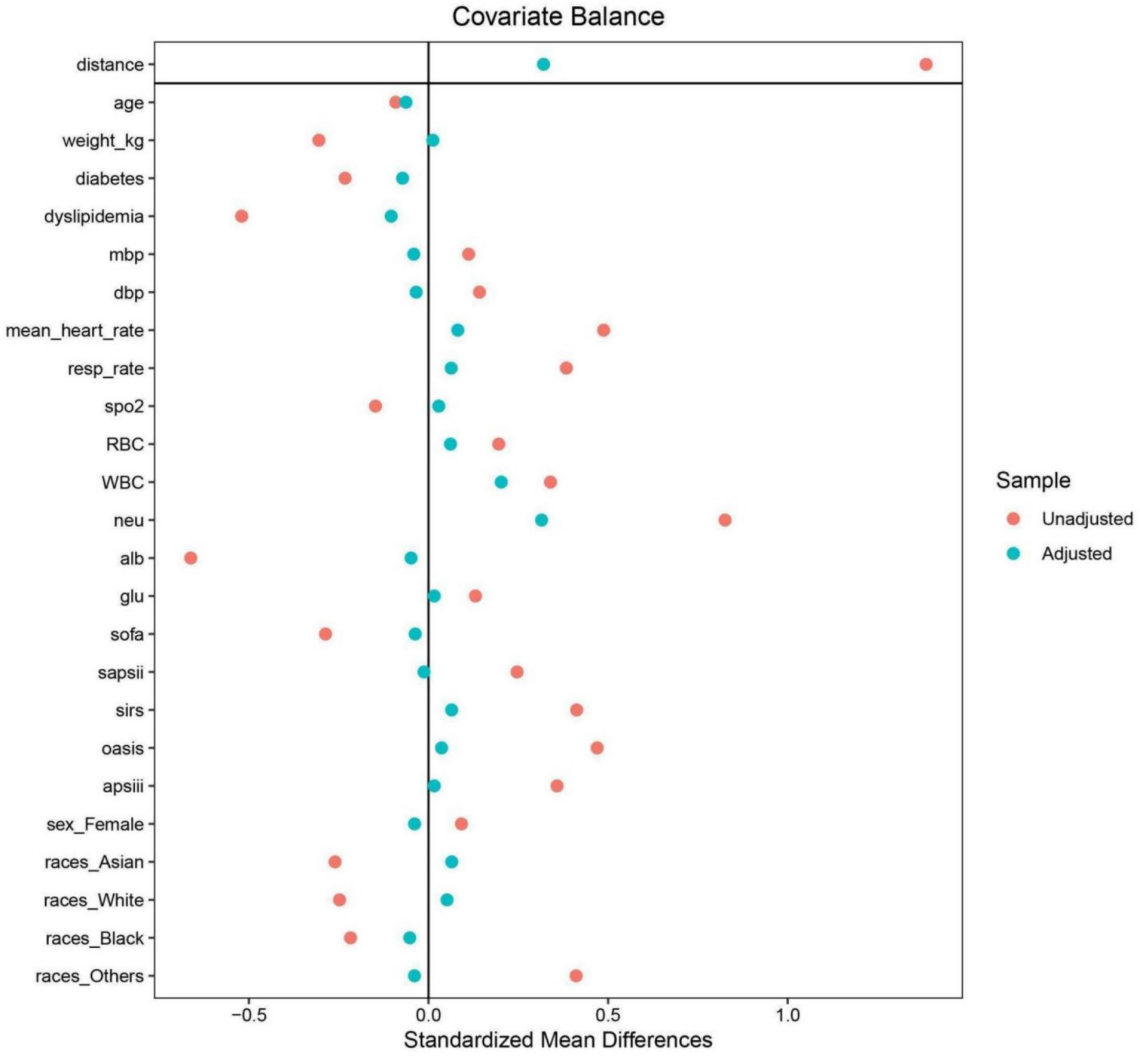
The absolute standardized differences for the matching variables between the two groups.

To mitigate baseline characteristic disparities between low and high ALI groups, a 1:1 PSM strategy was employed, culminating in the pairing of 199 patient dyads. Subsequent to PSM, a congruence in demographics, comorbidities, a majority of laboratory indicators, various metrics, and administered treatments was observed between the cohorts, as outlined in Table 3. The efficacy of the PSM was evaluated by the calculation of ASDs both pre- and post-PSM, with these results depicted in Figure 4.

**Table 3:** Baseline characteristics after PSM.

Variable	Overall (*n* = 398)	ALI		*p*-value
		<10.38 (*n* = 199)	≥10.38 (*n* = 199)	
*Demographics*				
Age, years	70 (60–80)	69 (56–79)	71 (61–80)	0.08
Men, *n* (%)	228 (57.29)	108 (54.27)	120 (60.30)	0.22
Ethnicity, *n* (%)				0.002
White	6 (1.51)	2 (1.005)	4 (2.010)	
Black	225 (56.53)	99 (49.75)	126 (63.32)	
Asian	33 (8.29)	13 (6.533)	20 (10.05)	
Others	134 (33.67)	85 (42.71)	49 (24.62)	
*Height, cm*				
Weight, kg	191 (47.99)	95 (47.74)	96 (48.24)	0.92
Comorbidities	122 (30.65)	55 (27.64)	67 (33.67)	0.19
Hypertension, *n* (%)	122 (30.65)	59 (29.65)	63 (31.66)	0.66
Diabetes mellitus, *n* (%)	171 (42.96)	74 (37.19)	97 (48.74)	0.02
Heart failure, *n* (%)	51 (12.81)	21 (10.55)	30 (15.08)	0.18
Cardiac arrhythmias, *n* (%)	43 (10.80)	24 (12.06)	19 (9.555)	0.42
Peripheral vascular disease, *n* (%)	217 (54.52)	89 (44.72)	128 (64.32)	<0.001
Chronic obstructive pulmonary disease, *n* (%)	41 (10.3)	18 (9.04)	23 (11.56)	0.41
Dyslipidemia, *n* (%)	6 (4–8)	6 (4–8)	7 (5–8)	0.04
*Prior stroke, n (%)*				
Charlson comorbidity index	1.68 (1.60–1.75)	1.68 (1.60–1.78)	1.68 (1.60–1.75)	0.61
Vital signs	75.5 (63.7–88.5)	73.5 (60.0–88.8)	77.0 (66.0–87.5)	0.12
Mean blood pressure, mmHg	86 (73–99)	88 (75–100)	82 (72–97)	0.03
Systolic blood pressure, mmHg	128 (109–147)	129 (111–147)	127 (108–147)	0.44
Diastolic blood pressure, mmHg	67 (57–80)	70 (59–84)	65 (54–78)	0.01
Mean heart rate, beats/min	84 (75–100)	88 (77–105)	80 (73–93)	<0.001
Respiratory rate, times/min	18 (16–22)	19 (16–24)	18 (15–21)	0.002
SpO_2_, %	99 (96–100)	99 (95–100)	99 (96–100)	0.72
*Laboratory parameters*				
Red blood cell, 10^9^/L	3.61 (3.04–4.19)	3.66 (3.13–4.22)	3.59 (2.99–4.11)	0.23
White blood cell, 10^9^/L	11.7 (8.3–15.4)	12.7 (9.6–16.9)	10.5 (7.4–13.7)	<0.001
Hemoglobin, g/L	11.0 (9.0–12.5)	10.9 (9.0–12.6)	11.0 (8.9–12.5)	0.82
Platelets, 10^9^/L	178.5 (131–234)	181 (133–261)	174 (129–218)	0.05
Neutrophil count, 10^9^/L	9.32 (6.42–13.03)	11.61 (8.74–14.97)	7.28 (4.94–9.95)	<0.001
Lymphocyte count, 10^9^/L	0.90 (0.63–1.25)	0.69 (0.52–0.99)	1.10 (0.87–1.41)	<0.001
Serum ALB, mg/dL	3.6 (3.0–4.0)	3.4 (2.8–4.0)	3.7 (3.3–4.0)	<0.001
Serum glucose, mg/dL	131 (109–168)	134 (112–174)	129 (104–157)	0.06
Serum sodium, mmol/L	138 (135–142)	139 (135–142)	138 (135–141)	0.38
Serum potassium, mmol/L	4.2 (3.8–4.6)	4.2 (3.8–4.6)	4.2 (3.8–4.8)	0.83
Serum creatinine	1.0 (0.7–1.3)	0.9 (0.7–1.3)	1.0 (0.7–1.3)	0.66
Anion gap, mmol/L	14 (12–16)	14 (12–16)	13 (12–15)	0.28
Prothrombin time, s	13.8 (12.1–16.0)	13.7 (12.1–15.7)	14.1 (12.2–16.2)	0.56
Activated partial thromboplastin time, s	29.5 (26.3–35.0)	28.7 (25.7–34.9)	30.6 (26.9–35.2)	0.06
ALI	10.33 (6.07–14.63)	6.07 (3.51–7.96)	14.63 (12.10–18.22)	<0.001
*Clinical severity scores*				
GCS	15 (15–15)	15 (15–15)	15 (15–15)	0.75
SOFA	1 (0–3)	1 (0–3)	1 (0–3)	0.14
SAPS-II	37 (30–46)	37 (30–48)	37 (30–45)	0.28
SIRS	34 (29–40)	35 (30–42)	32 (28–38)	<0.001
OASIS	42.5 (32–58)	44 (34–61)	40 (31–55)	<0.001
APS-III	15 (15–15)	15 (15–15)	15 (15–15)	0.01
*Treatment*				
Thrombolysis, *n* (%)	21 (5.28)	13 (6.53)	8 (4.02)	0.26
Thrombectomy, *n* (%)	20 (5.03)	15 (7.54)	5 (2.51)	0.02
*Clinical outcomes*				
LOS ICU, day	5.02 (2.28–9.95)	5.29 (2.82–9.95)	4.88 (2.03–10.23)	0.27
LOS hospital, day	12.79 (6.92–22.29)	12.88 (7.00–22.79)	12.25 (6.67–21.96)	0.74
ICU ACM, *n* (%)	57 (14.32)	40 (20.10)	17 (8.54)	<0.001
In-hospital ACM, *n* (%)	79 (19.85)	55 (27.64)	24 (12.06)	<0.001
30 days ACM, *n* (%)	94 (23.62)	67 (33.67)	27 (13.57)	<0.001
90 days ACM, *n* (%)	128 (32.16)	87 (43.72)	41 (20.60)	<0.001
1 year ACM, *n* (%)	155 (38.94)	100 (50.25)	55 (27.64)	<0.001

ALI, advanced lung cancer inflammation index; SpO_2_, saturation of pulse oxygen; ALB, albumin; GCS, Glasgow coma score; SOFA, sequential organ failure assessment score; SAPS-II, simplified acute physiology score-II; SIRS, systemic inflammatory response syndrome score; OASIS, Oxford Acute Severity of Illness Score; APS-III, acute physiology score-III; LOS, length of stay; ICU, intensive care unit; ACM, all-cause mortality; PSM, propensity score matching.

**Figure 5:**
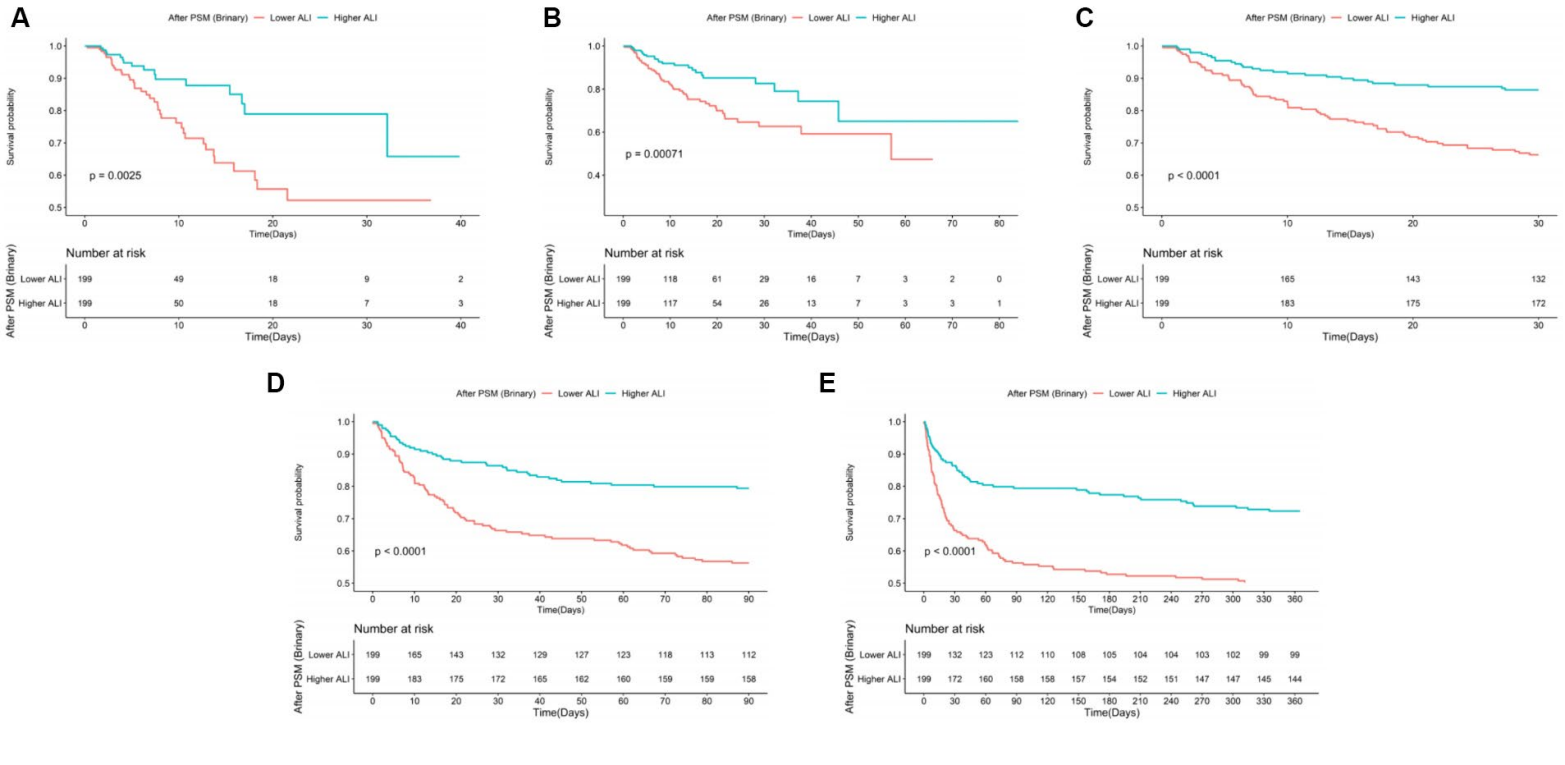
The K–M survival plots of **(A)** ICU, **(B)** in-hospital, **(C)** 30 days, **(D)** 90 days, **(E)** 1 year ACM categorized by binary of ALI after PSM. ACM, all-cause mortality; ALI, advanced lung cancer inflammation index; PSM, propensity score matching.

Following PSM, discernible disparities remained between the cohorts regarding ACM at various timeframes: in the ICU (20.10% vs. 8.54%, *p* < 0.001), during hospitalization (27.64% vs. 12.06%, *p* < 0.001), 30 days (33.67% vs. 13.57%, *p* < 0.001), 90 days (43.72% vs. 20.60%, *p* < 0.001), and 1 year (50.25% vs. 27.64%, *p* < 0.001). Differences in the length of stay in the ICU (LOS ICU) and hospital (LOS hospital), however, did not achieve statistical significance (*p* = 0.27 and *p* = 0.74, respectively). Furthermore, post-PSM multivariate Cox regression analysis affirmed that an ALI below 10.38 significantly forecasted increased ACM across the specified intervals: in the ICU (HR = 2.31, 95% CI: 1.27–4.21, *p* = 0.006), during hospitalization (HR = 2.16, 95% CI: 1.32–3.52, *p* = 0.002), and at 30 days (HR = 2.44, 95% CI: 1.54–3.86, *p* < 0.001), 90 days (HR = 2.25, 95% CI: 1.53–3.30, *p* < 0.001), and 1 year (HR = 2.10, 95% CI: 1.50–2.96, *p* < 0.001) benchmarks (as referenced in Table 4). K–M survival analysis further highlighted significantly lower survival rates for patients with an ALI <10.38 in comparison to those with an ALI >10.38 across both short- and long-term ACM evaluations, as evidenced in Figure 5.

**Table 4:** Cox regression analyses assessing the association between ALI and ACM in AIS patients after PSM.

Clinical Outcomes	Model 1		Model 2		Model 3	
	HR (95% CI)	*p*-value	HR (95% CI)	*p*-value	HR (95% CI)	*p*-value
**ICU ACM**						
ALI (>10.38)	Reference		Reference		Reference	
ALI (<10.38)	2.38 (1.33–4.25)	0.003	2.42 (1.34–4.37)	0.003	2.31 (1.27–4.21)	0.006
**In-hospital ACM**						
ALI (>10.38)	Reference		Reference		Reference	
ALI (<10.38)	2.24 (1.39–3.62)	0.001	2.11 (1.30–3.42)	0.003	2.16 (1.32–3.52)	0.002
**30 days ACM**						
ALI (>10.38)	Reference		Reference		Reference	
ALI (<10.38)	2.77 (1.77–4.33)	<0.001	2.55 (1.62–4.01)	<0.001	2.44 (1.54–3.86)	<0.001
**90 days ACM**						
ALI (>10.38)	Reference		Reference		Reference	
ALI (<10.38)	2.20 (1.58–3.06)	<0.001	2.16 (1.55–3.02)	<0.001	2.10 (1.50–2.96)	<0.001
**1 year ACM**						
ALI (>10.38)	Reference		Reference		Reference	
ALI (<10.38)	2.20 (1.58–3.06)	<0.001	2.16 (1.55–3.02)	<0.001	2.10 (1.50–2.96)	<0.001

Model 1: Unadjusted. Model 2: Adjusted age, gender, and ethnicity. Model 3: Adjusted age, gender, ethnicity, hypertension, diabetes, heart failure, thrombolysis, thrombectomy, white blood cell, red blood cell, systolic blood pressure, and SOFA. HR, hazard ratio; CI, confidence interval; ACM, all-cause mortality; ICU, intensive care unit; ALI, advanced lung cancer inflammation index; PSM, propensity score matching; AIS, acute ischemic stroke.

### Subgroup analysis for the ALI on both short- and long-term ACM in patients with AIS

**Figure 6:**
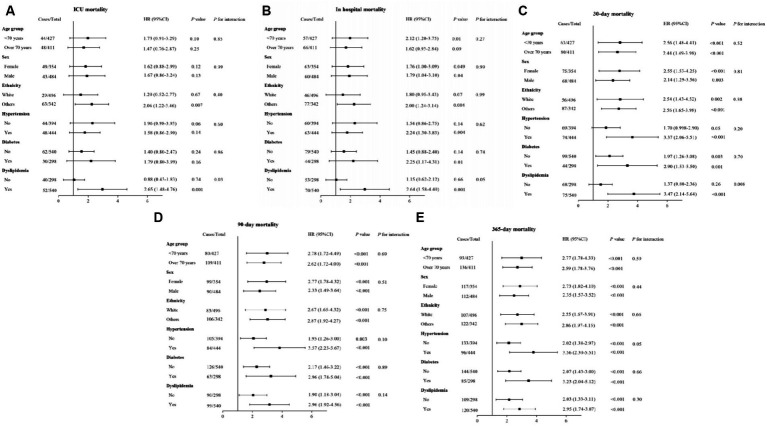
Forest plots of HRs for **(A)** ICU, **(B)** in-hospital, **(C)** 30 days, **(D)** 90 days, and **(E)** 1 year ACM in different subgroups. HR, hazard ratio; CI, confidence interval; ACM, all-cause mortality. HRs were adjusted for age, gender, ethnicity, hypertension, diabetes mellitus, heart failure, thrombolysis, thrombectomy, white blood cell, red blood cell, systolic blood pressure, and SOFA.

Subgroup analyses were performed to assess the impact of the ALI on both short- and long-term ACM among AIS patients, stratifying by demographic and clinical characteristics including age (<70 and ≥70 years), gender, ethnicity, presence of hypertension, diabetes mellitus, and dyslipidemia. These analyses consistently indicated an association between a lower ALI and increased risks of both short- and long-term ACM across nearly all examined subgroups, as depicted in Figure 6. Notably, the association between lower ALI and increased 30 days ACM did not reach statistical significance in the hypertension (*p* = 0.05) and non-dyslipidemia (*p* = 0.26) subgroups. Additionally, a significant correlation between lower ALI and higher ICU ACM was predominantly observed within the non-White (*p* = 0.007) and non-dyslipidemia (*p* = 0.001) subgroups. The interaction analysis did not demonstrate significant effects for short- and long-term ACM across most subgroups, with the exception of specific interactions in the dyslipidemia subgroup during ICU stay and at the 30 days mark (*p*_interaction = 0.03 and *p*_interaction = 0.008, respectively).

### Nonlinear relationship of ALI and both short- and long-term ACM

**Figure 7:**
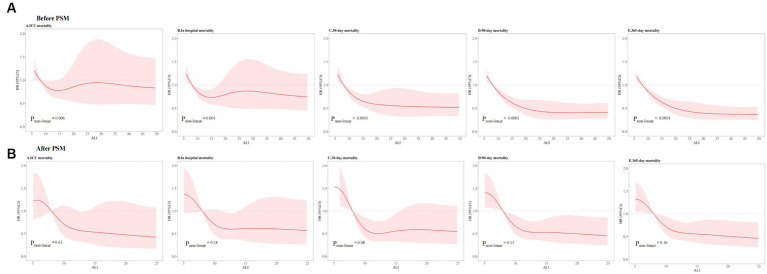
RCSs for ACM at different time intervals **(A)** before PSM and **(B)** after PSM. ACM, all-cause mortality; ALI, advanced lung cancer inflammation index; PSM, propensity score matching.

To investigate the presence of non-linear associations, RCS were utilized. Through the application of smooth curve fitting and generalized additive models, we examined the threshold effect exerted by ALI on ACM rates over both short- and long-term periods, aiming to pinpoint the inflection point. A linear relationship between ALI and both short- and long-term ACM was observable before PSM (ICU: *p*_non-linear_ = 0.006; hospitalization: *p*_non-linear_ = 0.001; 30 days: *p*_non-linear_ < 0.001; 90 days: *p*_non-linear_ < 0.001; 1 year: *p*_non-linear_ < 0.001). A non-linear relationship between ALI and mortality risk across various timeframes—namely, during the ICU stay, hospitalization, and at 30 days, 90 days, and 1 year intervals—was observable post-PSM (ICU: *p*_non-linear_ = 0.61; hospitalization: *p*_non-linear_ = 0.18; 30 days: *p*_non-linear_ = 0.08; 90 days: *p*_non-linear_ = 0.13; 1 year: *p*_non-linear_ = 0.16). The comprehensive statistical findings illustrating this correlation are presented in Figure 7.

## Discussion

In this research, we investigated the influence of the ALI on both short- and long-term ACM within a retrospective cohort derived from a nationally representative sample of AIS patients. Our findings initially revealed the distinct impact of ALI on ACM across AIS patients. Additionally, the study indicated that individuals exhibiting low levels of ALI faced an elevated risk of ACM, in contrast to those with higher levels of ALI. As far as we are aware, this investigation represents the inaugural effort to examine the implications of the ALI on ACM among AIS patients.

Extant literature has consistently illustrated the linkage between inflammatory and nutritional status and adverse clinical outcomes in patients with cerebrovascular disorders (4, 15–17). The principal finding of the present study underscores the independent and synergistic association of both inflammation and nutritional status with ACM in patients with AIS. Recent investigations deploying the ALI to evaluate clinical outcomes across a spectrum of cancer types have yielded heterogeneous results. An aggregate of six systematic reviews and meta-analyses elucidated the application of ALI in a variety of malignancies, including gastrointestinal cancers and NSCLC, demonstrating that ALI significantly influences cancer-related survival metrics, where a lower ALI is frequently associated with poorer prognoses (18–23). Consequently, this suggests the imperative for aggressive interventions in individuals presenting with diminished ALI. In the realm of hepatocellular carcinoma (HCC), research conducted by Wen et al. examined the relationship between ALI and both overall survival (OS) and cancer-specific survival following hepatectomy in HCC patients. Their findings advocate for the prognostic utility of ALI in predicting long-term survival outcomes, thereby underscoring its potential utility in the postoperative management of HCC patients (24). Thus, ALI emerges as a viable biomarker for preoperative evaluation. Furthermore, significant correlations have been established between ALI levels and clinical outcomes in non-cancer patient populations. Chen et al. (10), leveraging data from the NHANES database, explored clinical outcomes among patients with type 2 diabetes mellitus, revealing an association between reduced ALI levels and increased ACM and cardiovascular disease (CVD) mortality. Their analyses unveiled a J-shaped non-linear association for ACM and an L-shaped non-linear association for CVD mortality, emphasizing the criticality of maintaining ALI within an optimal range to ameliorate outcomes in type 2 diabetes mellitus patients through interventions like weight management and maintaining normal albumin levels. In the context of hypertension, analysis of the NHANES database identified ALI as an independent and significant prognostic factor within the American population (11), highlighting the paramount importance of evaluating and monitoring systemic inflammatory and nutritional status for the health maintenance of hypertensive individuals. Additionally, for patients with acute coronary syndrome (ACS) undergoing percutaneous coronary intervention (PCI), a lower ALI value was recognized as an independent prognostic risk factor, positing ALI as a novel marker for clinical application (12). Yuan et al. (13) discovered a significant association between higher ALI levels and reduced ACM and CVD mortality among elderly patients with heart failure, indicating ALI’s potential as a promising nutrition-inflammation marker with independent predictive value for assessing long-term mortality in this demographic. The insights derived from our study further affirm the relevance of ALI, particularly within the AIS patient cohort, as a robust biomarker for prognostication. Nevertheless, the applicability of ALI across other cerebrovascular diseases warrants additional investigation to fully ascertain its prognostic value.

Recent scholarly work has introduced a modified version of the ALI, defined as the product of the appendicular skeletal muscle index (ASMI) and serum ALB, divided by the NLR. The ASMI serves as a metric for evaluating muscle mass within the appendicular skeleton, encompassing the limbs, and is commonly applied in diagnosing sarcopenia, a condition characterized by the age-related decline in muscle mass and functionality. The computation of ASMI involves dividing the appendicular skeletal muscle mass (ASMM) by the height squared, with ASMM typically ascertained through imaging modalities such as magnetic resonance imaging (MRI), computed tomography (CT), or bioelectrical impedance analysis. Several studies have underscored the efficacy of the modified ALI in forecasting adverse outcomes across various patient populations, including those with lung cancer, renal cell carcinoma, and cancer cachexia (25–28). However, research led by Kim et al. (29), which examined the prognostic value of both the traditional ALI and its modified counterpart in patients with SCLC, revealed that the modified ALI, calculated using CT-determined lumbar 3 muscle index, did not offer prognostic advantages over the conventional ALI based on BMI. Given the complexity of its calculation, the traditional ALI, reflecting the nutrition-inflammation status more straightforwardly, may present a simpler and more broadly applicable prognostic tool.

Patients with AIS are notably susceptible to a concurrent state of malnutrition, inflammation due to immune deficiencies, and metabolic dysregulations. Therefore, the identification of a composite biomarker that encapsulates both nutritional and inflammatory dimensions is paramount. Distinguished from previous inflammatory markers, the ALI offers a holistic assessment of systemic status by integrating nutritional and inflammatory metrics, positing ALI as a potentially superior prognostic indicator compared to other biomarkers. The mechanistic underpinnings that might explain the observed reduction in mortality risk among AIS patients with higher ALI scores could be multifaceted. Firstly, ALI represents a calculated index, rather than a direct measurement, incorporating BMI, serum albumin levels, and the NLR as components reflective of nutritional and inflammatory states. BMI, a rudimentary metric of body adiposity, serves as a general gauge of nutritional health. Both spectrums of malnutrition—undernutrition and overnutrition—are partially discernible through BMI metrics. Notably, a substantial prospective study elucidated a J-shaped correlation between BMI and mortality risk, suggesting that an optimal, yet not excessive, BMI may signify better nutritional status, potentially bolstering immune function and mitigating malnutrition’s detrimental impacts (30). Secondly, serum albumin, a principal liver-synthesized protein, fulfills various roles, including the transportation and stabilization of nutrients, hormones, and medications (31). Recent research indicates that reduced albumin levels correlate with systemic inflammation activation and heightened malnutrition risk, while also highlighting albumin’s capacity to shield tissues from inflammatory damage (32). Thirdly, inflammation plays a pivotal role throughout all atherosclerotic plaque development stages, precipitating thrombotic incidents (33). Post-ischemic conditions witness the infiltration of circulating white cells into the brain and meninges, with neutrophils inflicting cerebral damage via the secretion of proteases, reactive species, and inflammatory cytokines (34). Conversely, lymphocytes serve as primary cerebral protective immunomodulators, crucial for inflammation-induced neuroprotection (35). More significantly, AIS incites systemic inflammation and neurohumoral pathway activations, potentially exacerbating immune dysregulation and impairing the functionality of peripheral organs, thus linking inflammatory markers to adverse AIS prognoses (36–43). In conclusion, sustaining a suitable body mass index, improved levels of serum albumin, and a decreased neutrophil-to-lymphocyte ratio can lead to an elevated advanced lung cancer inflammation index, which is associated with a more favorable prognosis.

### Strengths and limitations

When interpreting the findings of our study, it is imperative to consider both the strengths and limitations inherent to the research methodology employed. One of the significant strengths lies in the use of a nationally representative sample of U.S. patients with AIS, enhancing the generalizability of our results to broader populations. This approach facilitates rigorous analyses while accommodating a variety of confounders. Furthermore, the adoption of a 1:1 PSM method fortifies our findings by providing a robust mechanism for controlling confounders.

Despite these strengths, our study is subject to several limitations that warrant careful consideration: (1) the retrospective design and single database of the study inherently limits our ability to establish causality definitively. Although multivariate adjustments and subgroup analyses have been employed to mitigate confounding, the possibility of residual confounding cannot be entirely excluded; (2) this is particularly relevant for variables such as classifications based on the National Institutes of Health Stroke Scale, timing of stroke onset, and specific causes of death, which were not available within the database utilized for this study; (3) our investigation was limited to assessing the baseline ALI without the capacity to monitor its dynamic changes over the follow-up period. The absence of longitudinal data on ALI underscores the need for future research to evaluate the prognostic significance of ALI fluctuations; (4) our reliance on ICD codes for definitive diagnoses led to the exclusion of immediate complications such as stunned heart syndrome and pneumonia from our analysis. This exclusion could potentially inflate the observed ALI due to the severe exacerbation of cerebral perfusion and tissue necrosis; (5) significant advancements in stroke diagnosis and treatment from 2012 to 2019 could have influenced patient outcomes, highlighting the importance of considering these evolving standards of care and suggesting that future research should account for these changes to better understand their impact on prognosis; (6) ALI includes indicators which may be influenced by factors such as infection and stress, highlighting future research should aim to validate the applicability of ALI by more thoroughly excluding these factors. Recognizing these limitations is essential when evaluating the outcomes of our study. Future research endeavors should aim to validate and expand upon our findings, with particular emphasis on exploring the intricate relationships between nutrition, inflammation, and AIS. Investigating the limitations of using nutrition-inflammatory indices for the assessment of inflammatory conditions in AIS patients represents a critical avenue for further inquiry.

## Conclusion

In this retrospective cohort study, by utilizing a nationally representative sample of United States patients with AIS, our analysis elucidates a negative correlation between the ALI and ACM in individuals with AIS, underscoring the utility of ALI as a novel, efficacious, and accessible inflammatory biomarker for prognosticating ACM. These results carry profound implications for public health policy and practice. A deeper comprehension of these associations can empower public health practitioners and researchers to devise more targeted interventions and policies, aimed specifically at catering to the distinct needs of the AIS patient population, thereby enhancing their health outcomes. The further research in other races/ethnicity is urgent, particularly before applying these findings in clinical practice.

## Data availability statement

The original contributions presented in the study are included in the article/Supplementary material, further inquiries can be directed to the corresponding author.

## Ethics statement

Ethical review and approval was not required for the study on human participants in accordance with the local legislation and institutional requirements. Written informed consent from the patients/participants or patients/participants’ legal guardian/next of kin was not required to participate in this study in accordance with the national legislation and the institutional requirements.

## Author contributions

YH: Conceptualization, Data curation, Formal analysis, Investigation, Methodology, Software, Visualization, Writing – original draft, Writing – review & editing. XW: Conceptualization, Data curation, Methodology, Software, Writing – original draft, Writing – review & editing. ZL: Conceptualization, Data curation, Methodology, Software, Writing – original draft, Writing – review & editing. XY: Conceptualization, Data curation, Formal analysis, Funding acquisition, Investigation, Methodology, Resources, Software, Visualization, Writing – original draft, Writing – review & editing.
